# Comparative Analysis of the Effectiveness of the Topical Administration of Benzocaine and EMLA^®^ on Oral Pain and Tactile Sensitivity

**DOI:** 10.1155/2018/7916274

**Published:** 2018-02-07

**Authors:** David Gomes de Alencar Gondim, Antonio Marcos Montagner, Ivo Cavalcante Pita-Neto, Romildo José de Siqueira Bringel, Francisco Aurelio Luchesi Sandrini, Eduardo Fernando Chaves Moreno, Amanda Mendes de Sousa, Andreza Bastos Correia

**Affiliations:** ^1^São Leopoldo Mandic Institute and Research Center, Campinas, SP, Brazil; ^2^School of Dentistry, UNILEÃO-University Center, Juazeiro do Norte, CE, Brazil

## Abstract

**Objectives:**

To compare the effectiveness of the topical administration of benzocaine and EMLA on oral pain and tactile sensitivity.

**Materials and Methods:**

A randomized, double-blind, split-mouth clinical trial was carried out with 20 volunteers. The sensorial and quantitative tests were applied before the contact with topical anesthetic and after the application.

**Results:**

In the superficial tactile perception test, when we compared each group singly, there were statistically significant values in the decrease of superficial tactile perception when compared to the moment prior to the application of anesthetic agents. For the sensitivity to mechanical pain, no statistical significant difference was observed at evaluated times. In the needle penetration test, in an intergroup analysis, we found a decrease in the pain sensitivity to needle penetration at 5 min (*p*=0.053) and at 10 min (*p*=0.019) after the contact of the anesthetic drug with the oral mucosa.

**Conclusion:**

The application of topical anesthetic drugs reduces the discomfort associated with this procedure, mainly until the first 10 minutes. Only the needle penetration sensitivity test showed sufficient sensitivity to reveal a difference in the anesthetic effect between EMLA and benzocaine. This trial is registered with RBR-2N2GSW.

## 1. Introduction

Topical anesthesia is extremely important to a wide variety of dental procedures, such as periodontal probes, gingival manipulation, staple adaptation, preparation to infiltrative anesthesia, pediatric care, and traumatic lesions in the oral mucosa [1–3]. In this context, the main purpose of using topical anesthetic drugs is to reduce or relieve the painful stimulus caused by needle penetration, leading to significant control of pain and anxiety of the patient [1].

The ideal local anesthetic agent should be effective even when administered topically. However, not all anesthetic bases present this characteristic [4]. In dentistry, benzocaine is one of the main commercially available topical anesthetic drugs. It is chemically classified as an amino ester and is available as a gel. Although this drug presents rapid onset of action, it has limited potency and short anesthetic duration, besides being exclusively used in mucous membranes [5].

EMLA (AstraZeneca do Brasil, Ltda., Cotia, SP, Brasil) is an anesthetic formulation defined as an eutectic mixture of local anesthetic drugs composed of a combination of 2.5% prilocaine and 2.5% lidocaine. This formulation is indicated for pain control in several superficial cutaneous procedures [6, 7]. Accordingly, in the oral cavity, satisfactory results have been evidenced in biopsies [8], periodontal probes [9], and prior to local anesthesia [10].

Therefore, the aim of this study was to compare the effectiveness of the topical administration of benzocaine and EMLA on oral pain and tactile sensitivity.

## 2. Materials and Methods

Twenty-four academics from the dentistry course of Dr. Leão Sampaio University Center were interviewed in this study. Three of these academics were excluded because they did not attend the second visit and another because of diabetes and incomplete medical records. Thus, the study continued with 20 volunteers, including 12 women and 8 men with an average age of 22.8 years. The study was characterized as randomized, double-blind, split-mouth clinical trial.

The Research Ethics Committee of the Dr. Leão Sampaio University Center reviewed the procedures, including the recruitment and consenting process.

Healthy subjects (ASA I), meeting the criteria of the *American Society of Anesthesiology* (ASA) [11], were selected for this research, including nonpregnant, nonsmoker, nonnursing, with no history of allergies to local anesthetics, without chronic use of medications, with complete natural dentition and without reports of dysesthesia in the face or oral cavity. Participants who did not meet these criteria were excluded from the study.

**Figure 1:**
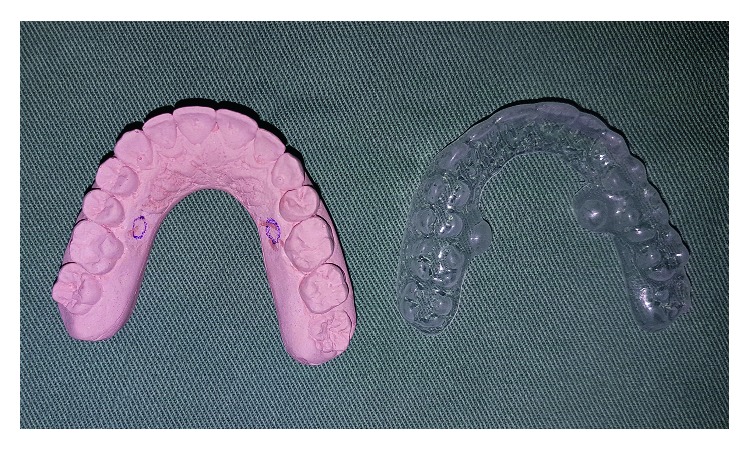
Acetate relief area for deposition of topical anesthetic drug.

Initially, we performed a molding of the maxillary arch of the participants, followed by the preparation of a model in plaster and acetate molding, with involvement of the teeth and palate. A relief area was created in wax and placed on the cast model on the palate, at 2 mm from the gingival margin, between the teeth 15 and 16 and 25 and 26, to create a reservoir for the local anesthetic drug (Figure 1). The participants of this research were comfortably seated in the dental chair at 45° in relation to the ground, with the head resting on a flat surface, in a silent room with temperature set at 23°C. They were instructed to keep their eyes closed and concentrate on the examination.

**Figure 2:**
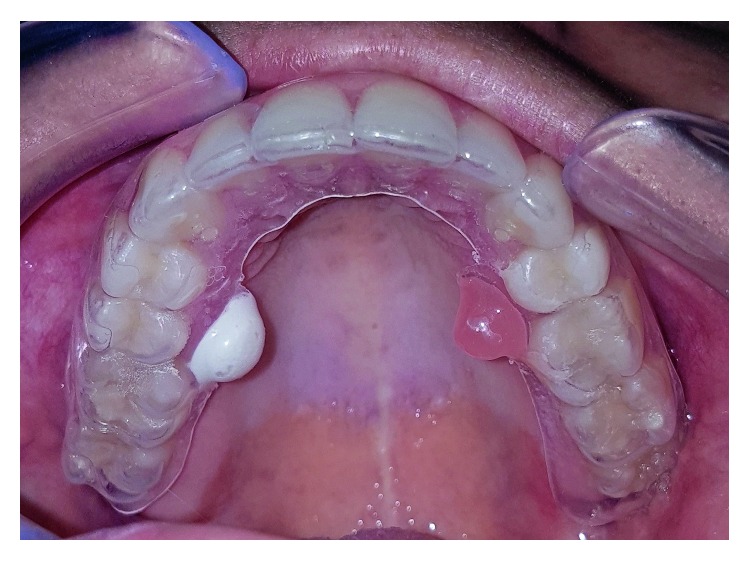
Acetate splint loaded with topical anesthetic.

We applied 0.4 g of 20% benzocaine (DFL Industria e Comércio SA, Rio de Janeiro, RJ, Brazil) to the reservoirs, and in the opposite portion, we deposited an equivalent quantity of EMLA (AstraZeneca do Brasil, Ltda., Cotia, SP, Brazil) (Figure 2). The sides of deposition of the topical anesthetic were randomly selected, by sorting a sealed envelope. The mucosa was previously dried with gauze, and then the tray was positioned and maintained for 5 min. Subsequently, the excess anesthetic was removed from the teeth and gingiva, and the area was dried with air jets to prevent changes in sensitivity and/or sliding of the needle or filaments, which could result in misinterpretations of the examinations.

An examiner who was not present in the previous period was responsible for assessing the values related to the pain stimulus, which consisted of three quantitative sensory methods: superficial tactile perception, sensitivity to mechanical pain, and sensitivity to needle penetration. The tests were applied at the same previously identified points, beginning from the patient's right side to the left and sequentially repeated for three times each test.

### 2.1. Superficial Tactile Perception

This parameter was evaluated through the application of Semmes–Weinstein microfilaments (Sorria-Bauru, Bauru/SP, Brazil), consisting of a set of 7 nylon filaments with equal lengths, rounded tips, and different diameters and colors, which allows the application of forces of different intensities (0.05 to 300 g/mm^2^) on a surface. The filaments were vertically applied at the predetermined points, in ascending order of diameter, until perception and identification of the stimulus by the participant. The filament in which the stimulus was perceived was recorded. Of note, three measurements were taken for each side.

### 2.2. Sensitivity to Mechanical Pain

The sensitivity to mechanical pain was determined through application of a constant force using the orange-colored Semmes–Weinstein filament (which corresponds to a force of 10 g/mm^2^) in contact with the mucosa, for 2 seconds. The evaluated patient quantified the level of pain through the visual analogue scale (VAS) [12], which assigns a value of 0 for “complete absence of pain” and 10 for “the most intense possible pain level.” Three measurements were made for each side.

### 2.3. Sensitivity to Needle Penetration

A short 30G dental needle (DFL Industria e Comércio SA, Rio de Janeiro, RJ, Brazil) was vertically inserted at the points that had contact with the topical anesthetic drugs, at a depth of 2 mm, controlled by means of a rubber stopper. The evaluated patient quantified the level of pain through the visual analogue pain scale [12] as previously described. Three measurements were taken for each side. The measurements started before the tray was applied and after contact with the topical anesthetic drugs at 5, 10, 20, and 30 min.

## 3. Statistical Analysis

The data were examined and classified as nonparametric and nonnormal, after application of the D'Agostino's test, and median values were applied in the three evaluation methods. For intergroup analysis (Benzocaine x EMLA), the Wilcoxon test was applied. To perform comparisons in the same group, according to the time of contact with the topical anesthetic drug, the Friedman test was applied. All time points (5, 10, 20, and 30 min) were assessed, and odds below 0.05 were classified as statistically significant. All data were evaluated using the SPSS (SPSS, Windows version 19.0, SPSS Inc., USA).

## 4. Results

**Figure 3:**
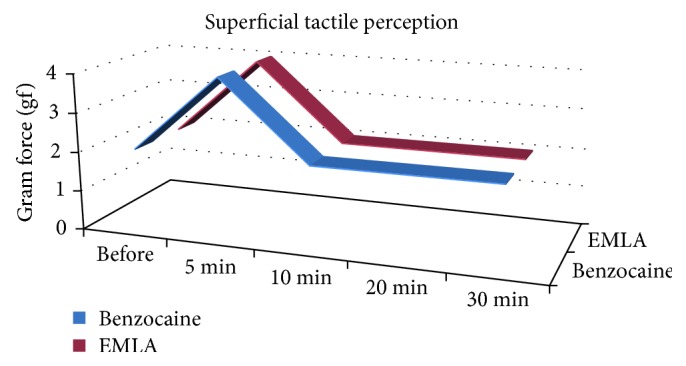
Median values for superficial tactile perception of benzocaine and EMLA according to the evaluated times and exerted force.

In the superficial tactile perception test, we observed a similar result between the side treated with EMLA and the side that was treated with benzocaine, represented by a decrease of tactile response to higher pressures in the oral mucosa in the five minutes time point, obtained with the use of thicker *Semmes–Weinstein* filaments (Figure 3). However, when comparing both groups (EMLA versus benzocaine), no statistical repercussions were observed at the evaluated times (Table 1).

When we compare each group singly, we observed that, at the five minutes point of measurement, there were statistically significant values in the decrease of superficial tactile perception when compared to the moment prior to the application of anesthetic agents. These results were not statistically significant for the other times (10, 20, and 20 minutes) (Table 2).

**Figure 4:**
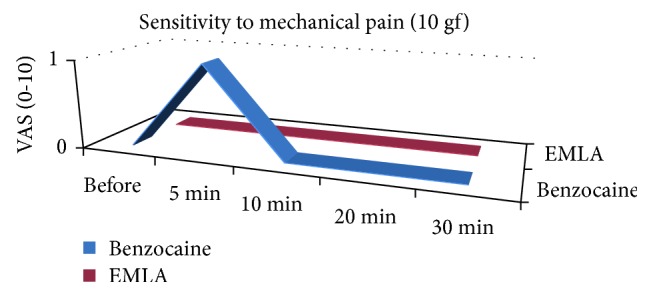
Median values for sensitivity to mechanical pain of benzocaine and EMLA treatments, according to the evaluated times and visual analogue scale (VAS).

The test of sensitivity to mechanical pain revealed that the patients presented minimal discomfort as attested by the analogue scale [12]. A comparative analysis between the treatments with the two drugs demonstrated a slight increase in the mechanical sensitivity in the benzocaine-treated side (Figure 4), at five minutes point. However, no statistical significant difference was observed at evaluated times, either in the intergroup analysis (Table 1) or in an isolated group assessment (Table 2).

**Figure 5:**
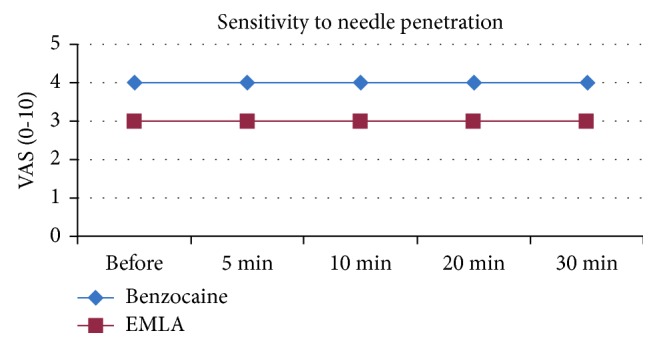
Median values for sensitivity to needle penetration of benzocaine and EMLA treatments, according to the evaluated times and visual analogue scale (VAS).

In the test of sensitivity to needle penetration, the benzocaine-treated side was more sensitive to the painful stimulus when compared to the EMLA-treated side, evidenced by higher visual analogue pain scores (Figure 5). In an intergroup analysis, we found a decrease in the pain sensitivity to needle penetration at 5 min (*p*=0.053) and 10 min (*p*=0.019) after the contact of the anesthetic drug with the oral mucosa (Table 1), with statistical relevance. When we evaluated each group separately, we observed that the side in contact with benzocaine had a statistical difference in point time of 30 minutes (Table 2), when compared to the time prior to contact with the topical anesthetic. No other information with statistical relevance was observed for the other time evaluated and in the group treated with EMLA.

## 5. Discussion

Dental anesthesia is among the main procedures associated with patient phobia in dental offices, leading a considerable number of adults to avoid dental treatments because of the fear of anesthetic puncture [13]. Nevertheless, patients with anxiety due to aversion to dental procedures usually report greater pain sensation during anesthesia than those who do not fear the anesthetic act [14].

Topical anesthesia has a main objective to annul pain prior to anesthetic infiltration [15]. This procedure optimizes infiltrative local anesthesia by reducing the level of anxiety of the patient before needle penetration, as well as decreasing the number of perforations required and the amount of anesthetic administered [2].

With the exception of the needle penetration sensitivity test, the other instruments of evaluation were not sensitive enough to show a statistical difference between EMLA and benzocaine, unlike other reports that revealed clearer results on the superiority of EMLA [10, 16].

The statistically significant results in superficial tactile perception test, on both sides, when evaluated singly (Table 2), may reflect a blockade of A*β* fibers and mechanoreceptors. Although it was not possible to measure the intensity of this blockade, previous studies have demonstrated that topical administration of 5% lidocaine interferes with the fibers that are responsible for touch perception, which justifies the maintenance of touch sensitivity in some patients, even after contact with the topical anesthetic drug [17].

The influence of anesthetic bases on nociceptors and C fibers, both associated with pain stimuli, were evaluated by the tests of sensitivity to mechanical pain and needle penetration sensitivity. The first test was not sensitive enough to confirm statistical differences between EMLA and benzocaine in the two modalities of evaluation (intergroups and isolated). In our study, the VAS was used only to measure the intensity of pain. As observed in others reports [18]. if we had used it to quantify both degree of discomfort and pain, it might reflect more consistent values.

In line with other studies [16], in the needle penetration sensitivity test, the side in contact with EMLA revealed less pain stimulation (Figure 5). When we correlated the repercussions of the anesthetic effect over the evaluated times (5, 10, 20, and 30 min), statistically significant outcomes were obtained in the first 5 minutes and 10 minutes after contact with the topical anesthetic, when the needle penetration sensitivity test was applied (Table 1). These results support the evidence that local anesthetic drugs affect more the pain threshold than the sensory threshold [19].

Although we did not perform an evaluation of the time of action of the evaluated anesthetics, the main results with statistical relevance were present in the first 5 and 10 minutes of contact. This fact is corroborated by other studies that reveal duration of anesthetic effect over 20 minutes for EMLA [18].

To achieve a consistent evaluation of the topical anesthetic action, the methods of measurement should include not only the response to pain through the visual analogue scale, but also the influence on the somatosensory system, evaluated in this study by the superficial tactile perception and corroborated by other works that demonstrated favorable results for EMLA in the effectiveness of changing sensory and pain thresholds [20]. Thus, the use of Semmes–Weinstein needles and filaments in the measurement of the sensitive and painful responses in the present study were effective and simple to acquire and apply. Accordingly, earlier reports ratified the reliability and validation of these instruments for use in the oral cavity. However, electronic measurements have showed greater precision in the results [18, 20].

In contrast to benzocaine, which has specific characteristics for use in the mouth, the prilocaine/lidocaine combination (EMLA) used in this study has properties that impair its use in the oral mucosa. In fact, the unpleasant taste was one of the complaints of the participants, and the addition of flavor would facilitate their acceptance, especially in pediatric dentistry. In addition, the low viscosity of this product makes difficult its administration on the palate, requiring the formulation of a tray with a specific reservoir. Although better flavored formulations containing lidocaine/prilocaine with specific applicators are currently available, their use is restricted to periodontics and they have limited scope [9].

Reports of local or systemic adverse effects of topical anesthetic drugs are uncommon [21] and were not evidenced in this study. The absorption and bioavailability of the drug depend on the contact surface, concentration, and time of application. Therefore, the use of these drugs in integral surfaces, for a brief time and at a low dose, as used in this work, is relatively safe. Accordingly, in this study, we used only 1/4 (0.4 g) of the recommended maximum dose (2 g) of benzocaine and 1/10 of the dose that achieves plasma levels that are toxic to the central nervous system [22, 23].

Finally, we conclude that the application of topical anesthetic drugs prior to anesthetic infiltration in the oral mucosa reduces the discomfort associated with this procedure, mainly until the first 10 minutes. Only the needle penetration sensitivity test showed sufficient sensitivity to reveal a difference in the anesthetic effect between EMLA and benzocaine. An improvement in the other two methods, associated with an increase in the number of participants, may provide a more complete analysis of the both topical anesthetics.

## Figures and Tables

**Table 1:** Intergroup comparative analysis according to the methodological test and *p* value for each time point.

Sensitivity to mechanical pain (10 gf)				
Benzocaine versus EMLA				
Time point (min)	5	10	20	30
*p* value	*p*=0.96	*p*=0.76	*p*=0.15	*p*=0.18
Sensitivity to needle penetration				
Benzocaine versus EMLA				
Time point (min)	5	10	20	30
*p* value	*p*=0.053^∗^	*p*=0.019^∗^	*p*=0.31	*p*=0.67
Superficial tactile perception				
Benzocaine versus EMLA				
Time point (min)	5	10	20	30
*p* value	*p*=0.2	*p*=0.6	*p*=0.17	*p*=1

^∗^ *p* < 0.05 showing significant difference between EMLA and benzocaine.

**Table 2:** Comparison in the same group according to the methodological test and *p* value for each time point.

Sensitivity to mechanical pain (10 gf)				
Benzocaine side				
Time point (min)	5	10	20	30
*p* value	*p*=0.50	*p*=0.99	*p*=0.99	*p*=0.99
EMLA side				
Time point (min)	5	10	20	30
*p* value	*p*=0.99	*p*=0.99	*p*=0.99	*p*=0.72
Sensitivity to needle penetration				
Benzocaine side				
Time point (min)	5	10	20	30
*p* value	*p*=0.28	*p*=0.43	*p*=0.15	*p*=0.02^∗^
EMLA side				
Time point (min)	5	10	20	30
*p* value	*p*=0.06	*p*=0.21	*p*=0.49	*p*=0.23
Superficial tactile perception				
Benzocaine side				
Time point (min)	5	10	20	30
*p* value	*p*=0.01^∗^	*p*=0.69	*p*=0.43	*p*=0.75
EMLA side				
Time point (min)	5	10	20	30
*p* value	*p*=0.04^∗^	*p*=0.13	*p*=0.41	*p*=0.73

^∗^ *p* < 0.05 showing influence of the topical anesthetic in each test.
